# The use of the Baumber scoring system for metastatic disease of the vertebral column

**DOI:** 10.1186/s13104-025-07390-1

**Published:** 2025-07-22

**Authors:** Sam Hodgson, Paul Pynsent, Simon Hughes, Petr Rehousek, Adrian Gardner

**Affiliations:** 1 Institute of Clinical Sciences, University of Birmingham, Edgbaston, Birmingham, B15 2TT UK; 2 The Royal Orthopaedic Hospital NHS Foundation Trust, Bristol Road South, Northfield, Birmingham, B31 2AP UK; 3 Aston Medical School, Aston University, Birmingham, B4 7ET UK

**Keywords:** Metastasis, Spine, Tumour, Survival, Prognosis

## Abstract

**Objective:**

The prognostic assessment of metastatic spinal disease is predominantly tumour, rather than patient based. In 2021, Baumber et al. published a prognostic scoring system based on the patient as a whole rather than the tumour within the patient for metastatic disease of the appendicular skeleton. This paper assesses that prediction formula in those with metastatic disease of the spine.

**Results:**

Survival was recorded for 65 individuals who underwent surgery for spinal metastatic disease. Using the same parameters and hazard ratio as Baumber, the projected survival was longer than actually occurred (over-prediction of 39% at 6 months and 54% at 12 months). The relative contributions of the individual parameters as part of the overall survival was different between the groups with a greater contribution seen if the individual had hyponatraemia, hypoalbuminaemia and low levels of creatinine. The reasons for the differences seen between the spinal and appendicular groups with regards to these parameters are not clear but may represent a poorer level of general health or the behaviour of different types and subtypes of malignancy. Further work is required to develop a specific tool for the calculation of prognosis in a metastatic spinal cohort using a general health perspective.

## Introduction

Metastatic spread of cancers to the spine is a growing healthcare problem [1]. From the clinical perspective, vertebral body bony destruction by malignancy leads to instability of the vertebral column which leads to pain and the inability to mobilise, along with spinal cord and nerve root dysfunction caused by compression of the neural structures leading to neurological disability [2]. The clinical decision required in the management of metastatic spinal disease is whether or not surgical intervention is both indicated from the spinal point of view and holistically, whether it is in the patient’s best interests, when assessing the risks and overall prognosis. Scoring systems used to predict the prognosis from metastatic spinal disease are reported in the literature [3–9]. The Tokuhashi system [8] is recommended by the National Institute of Clinical Excellence (NICE) [2] for the management of metastatic spinal cord compression in the UK. The Tokuhashi system is a series of assessments of the patient including the general condition (as scored using the Karnofsky Performance Status), the number of extra spinal bone metastases, the number of metastases in the vertebral body, the presence of metastases to the major internal organs, the site of primary cancer and an assessment of spinal cord function using there Frankel grade [8]. As such the Tokuhashi score is based around the tumour, rather than the generalised patient condition.

More recently, a prognostic scoring system for survival following surgery for metastatic disease of only the appendicular skeleton was developed by Baumber et al. [10] with the structure:

% Risk = [1-(K^α^)] × 100, where α is the summed hazard ratios and K is a constant for survival at either 6 months (0.94516) or 12 months (0.90619). This scoring system was different to previous scoring systems for the appendicular system such as the Mirels score [11] in that Mirels like Tokuhashi [8] really only assesses the lesion rather than as a holistic assessment of the patient. The Baumber system [10] however assesses the patient as a series of interlinked body systems (ASA grade, white cell count, sodium level, heart rate and primary tumour type) that form a multivariable analysis for predicting survival. In their paper, Baumber et al. [10] specifically excluded spinal disease from their patient cohort because of the presence of previous scoring systems for spinal metastatic disease already in the literature. Consequently, it is not known whether the findings of Baumber et al. [10] with regards the markers of body system health and survival after metastatic long bone disease are applicable to spinal metastatic disease.

This paper will address this by applying the findings of Baumber et al. [10] to assess the utility of the system in a cohort of patients with metastatic spinal disease, as compared to the original appendicular skeleton.

## Main text

### Background and ethical considerations

This is an analysis of routine care data collected as part of the emergency service offered by a supra-regional specialist spinal centre for the management of metastatic spinal disease. All the individuals in this data underwent an operative intervention. This study was given ethical and research approval by the Health Research Authority (HRA) and Health and Care Research Wales (22/HRA/1507). As this was a retrospective analysis of already collected clinical data, and that there was never going to be any contact made with any of the patients in the study, it was deemed that informed consent would not be necessary to perform this work.

## Methods

The data analysis followed the methods described by Baumber et al. [10] with respect to the parameters and all data were analysed using the R statistical platform version 4.3.2 [12]. Specifically the survival of the cohort was modelled using the R survival analysis software [13]. The hazard ratios for the parameters specific to the spinal cohort were calculated and compared to those of the original Baumber cohort to predict survival for the spinal cohort at 6 and 12 months following operative intervention.

## Results

Between June 2019 and March 2022, there were 65 individuals referred who underwent some form of surgical intervention for metastatic spinal disease consisting of 31 males and 33 females with a mean age of 64 years (standard deviation 12 years, range 31 to 85 years). The primary malignancies were lung (n = 17), breast (n = 9), prostate (n = 9), gastrointestinal (n = 7), renal (n = 6) and others (endometrial, haematogenous and sarcoma) (n = 9). There were also cases where the primary malignancy was unknown at time of surgery (n = 8). All cases underwent a surgical reconstruction with instrumentation apart from four, who underwent a vertebroplasty procedure only.

**Fig. 1:**
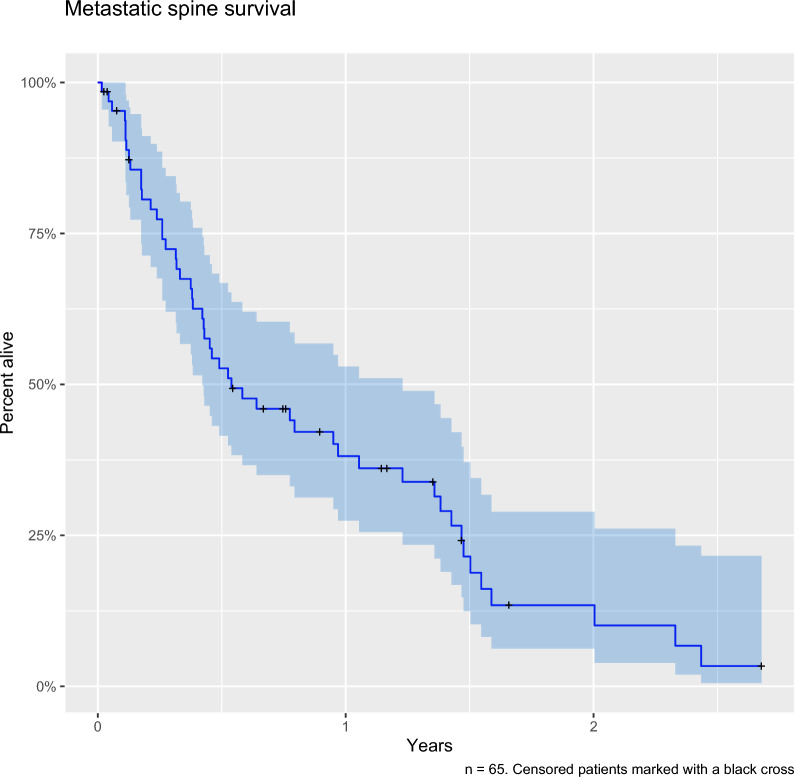
Survival curve of the surgical cohort

The survival curve for the surgical cohort is shown in Fig. 1. The range of survival was from a minimum of 6 days to a maximum of 978 days. There were 49% (n = 32) of individuals still alive at 6 months and 30% (n = 19) at 12 months.

**Fig. 2:**
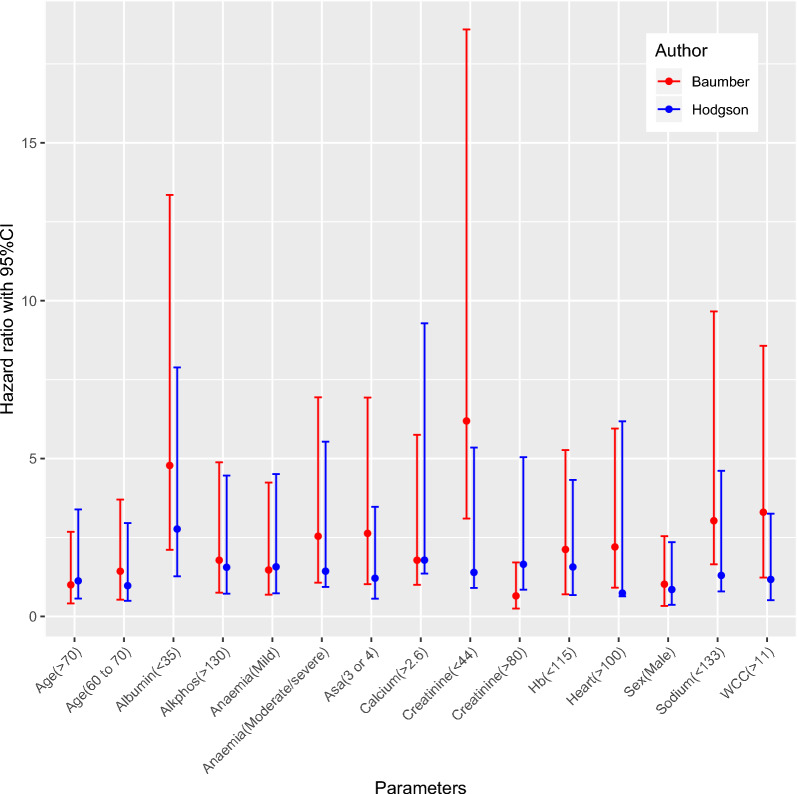
A comparison of the hazard ratios and 95% confidence intervals of the parameters from the Baumber et al. [10] and those calculated from the spinal data

**Fig. 3:**
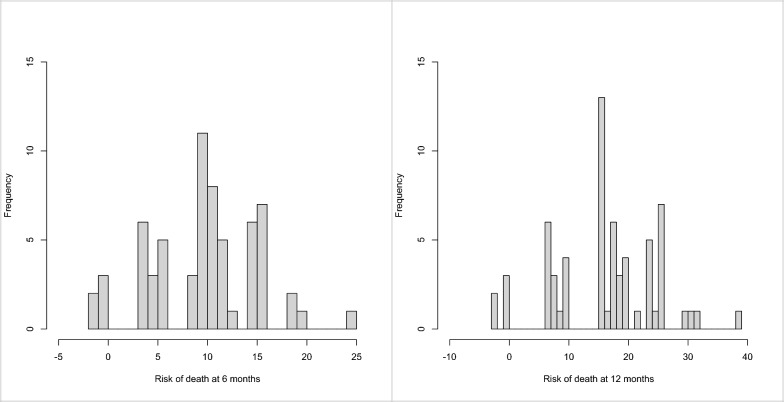
Individual risk of death at 6 and 12 months calculated using the formula for Baumber et al. [10]

The distribution of the hazard ratios (and the 95% confidential intervals of that hazard ratio) for the spinal cohort are demonstrated against those from Baumber et al. [10] in Fig. 2. Of interest, there is a difference between the hazard ratios derived from the patient group of the Baumber paper [10] and the patients in this spinal cohort, particularly seen for the parameters of Albumin (< 35), Creatinine (< 44), Sodium (< 133) and White Cell Count (> 11). Using the same formula with the listed parameters and weighted hazard ratios as in the paper by Baumber et al. [10], the mean survival of the spinal cohort was predicted to be 90% at 6 months (95% CI of 89% and 92%) and 84% at 12 months (95% CI of 82% and 87%) (10% risk of death at 6 months and 16% at 12 months). This was an over-prediction of survival when compared to what actually happened of 39% at 6 months (p < 0.0001) and 54% at 12 months (p < 0.0001). Histograms of the individual risk of death using the same formula and weighted coefficients as in the paper by Baumber et al. [10] at 6 and 12 months are seen in Fig. 3.

## Discussion

Metastatic spinal disease, as a consequence of an ageing population who are benefitting from a more successful survival from the treatment for their primary neoplastic disease, is a growing issue for healthcare providers [1]. Providing evidence based information to individual patients about their condition, and their specific prognosis following the different options for treatment that exist is key to maximising good outcomes and minimising morbidity and mortality as a consequence of that intervention. Prognostic scoring systems, developed following the analysis of key parameters of health and disease in large numbers of patients with metastatic spinal disease, are a method of quantifying risk and prognosis on an individual basis. This has been done in a number of formats in the literature [3–9].

Outside of metastatic spinal disease, Baumber et al. [10] approached prognostic prediction in a different fashion, specifically for metastatic disease of the appendicular skeleton using a patient organ system approach, similar in nature to a standard assessment of fitness for general anaesthesia, rather than an assessment based on the burden of the malignancy. This approach was used based on the assessment that the physiological state of the patient, along with their co-morbidities, could be of more use in prediction of outcome than an assessment of the tumour and associated tumour burden. Using this approach, a number of significant parameters (with associated hazard ratios) that could be used to predict survival at 6 and 12 months were generated. In their paper, Baumber et al. [10] specifically excluded patients with spinal metastatic disease because of the presence of other scoring prognostic systems.

This paper reproduces the methodology used by Baumber et al. [10] in a cohort of individuals with metastatic spinal disease, from a number of different primary malignancies, who underwent surgery on their spine to assess whether a similar approach may have utility in this group. Our results has found that the predicted survival using the Baumber methodology following surgery for metastatic spinal disease is not as good as that reported for metastatic disease in the appendicular skeleton. As a consequence of this, the survival prediction of the Baumber tool is not as accurate for spinal disease as for limb disease with an over prediction of survival by 39% at 6 months and 54% at 12 months. It is difficult to identify why certain parameters and hazard ratios derived from the patient group of the Baumber paper [10] and from the spinal cohort, particularly seen for the parameters of Albumin (< 35), Creatinine (< 44), Sodium (< 133) and White Cell Count (> 11) are more influential in the spinal cohort when compared to that of metastatic disease in the appendicular skeleton. Inferences that could be drawn here are that individuals with spinal metastatic disease are generally less well overall, and thus have less resilience and reserve to derangements of other organs in the body. This is indicated by the effects of hyponatraemia, hypoalbuminaemia and low creatinine levels, which are all markers of global poor health. It is also possible to infer that the primary disease that metastases to the spine is different to that metastasising to the appendicular skeleton, both in subtypes of the same primary disease site of primary [14, 15] or as different primary cancers [16], and a feature of this could be related to the debilitating nature of that on the individual as a whole. However, it does also raise questions about the generalisability of the work of Baumber et al. away from the appendicular skeleton to the spine [10] because of the number of similarities between metastatic disease of limbs and spine when assessed in a holistic, non-skeletal, body organ fashion, and it may be reasonable to expect there to be a closer relationship. This work demonstrates that there is promise in the prediction of survival from metastatic cancer in both the limbs and spine using a holistic, body system approach, but there is a need to further investigate the most appropriate parameters in each scenario which would then be validated in appropriate populations of sufficient size to create a robust model.

## Conclusion

Baumber et al. [10] published a method of assessing the risk of death from appendicular skeletal metastatic disease which, when applied to metastatic spinal disease, over-estimates the true survival over time. The reasons for this are not clear, but there is some utility in the Baumber approach in metastatic spinal disease. Future work will address this.

## Limitations

This work has limitations. It is based on the work by Baumber et al. [10] which is not validated in a population outside of the authors institution and so there is a query over the generalisability of their work in the first instance. This paper has applied their work to a different population with the same pathology at a different anatomical location, but using the same hazard ratios. A larger sample size than currently available would be required to be able to validate the original hazard ratios, or to calculate new hazard ratios, for this spinal population.

## Data Availability

The datasets used and/or analysed during the current study are available from the corresponding author on reasonable request.

## Abbreviations

ASA: American Society of Anesthesiologists
NICE: National Institute for Clinical Excellence
HRA: Health Research Authority
UK: United Kingdom
WMA: World Medical Association
